# Effect of Punch Geometry on Stress and Strain Distribution During Contact Lens Demolding

**DOI:** 10.3390/mi17010010

**Published:** 2025-12-22

**Authors:** Ching-Mu Cheng, Yun-Shao Cho, Tieh-Fei Cheng, Jui-Yu Wang, Jung-Jie Huang

**Affiliations:** 1 Department of Electrical Engineering, Da-Yeh University, Changhua 515006, Taiwan; chengcm458045@gmail.com; 2 Henghau Enterprise Company Limited, Hsinchu 303117, Taiwan; tfc1973@gmail.com; 3 Graduate School of Engineering Science and Technology, National Yunlin University of Science and Technology, Yunlin 640301, Taiwan; bjo4m4wang@gmail.com; 4 Department of Computer Science and Information Engineering, Asia University, Taichung 413305, Taiwan

**Keywords:** contact lens, demolding stress, punch-assisted demolding, finite element analysis, process yield

## Abstract

This study optimized the punch-assisted demolding technique for the separation of contact lenses, incorporating finite-element analysis to evaluate the effects of punch geometry (punch material: 304L stainless steel) on the stress and strain distributions of polypropylene lens molds. The simulation results revealed that the punch surface featured a flat base with a central arc-shaped groove (groove diameter: 7 mm, depth: 0.75 mm), which exhibited optimal stress dispersion characteristics during the demolding process, effectively reducing mold deformation. Experimental validation over 100 demolding cycles confirmed that the use of the aforementioned punch resulted in the manufactured lens having high central stability and reduced van der Waals forces during demolding, allowing smoother lens release and facilitating improved demolding performance. Comprehensive evaluation based on defect inspection and centering stability indicated that a yield of 82% was achieved with the optimized punch, with this yield being 13% higher than that obtained with a flat punch lacking an arc groove (69%). These results indicate that the optimized punch design not only reduces development costs but also enhances manufacturing yield and throughput, demonstrating strong potential for application in contact lens production.

## 1. Introduction

The increasing ubiquity of screens, in smartphones and other devices, has led to worsening vision becoming a widespread public health concern. In particular, myopia and hyperopia impair everyday functioning and burden health-care systems [1,2]. According to the World Health Organization, more than half of the global population is affected by some degree of refractive error, and by 2050, the number of people with myopia is projected to exceed five billion, posing a major public health challenge [3,4].

Contact lenses have become crucial alternatives to traditional glasses. Compared with traditional glasses, contact lenses offer a wider field of view and greater freedom of movement, making them especially suitable for specific groups, such as athletes, performers, and individuals requiring long-term wear [5,6]. With advancements in polymer materials and molding technologies, traditional hydrogel-based contact lens manufacturing has reached a relatively mature stage, offering advantages such as process simplicity, stable mass production, and low material cost. Hydrogel contact lenses are typically created using hydrophilic monomers (e.g., 2-hydroxyethyl methacrylate) that exhibit high moldability and stability after their polymerization, making them suitable for large-scale automated production [7]. However, after the hydrogel material is formed within the mold, molecular physical attraction arises between the two, resulting in the presence of van der Waals forces, causing adhesion during molding and demolding. This phenomenon often results in incomplete lens edges, surface defects, or fractures, thereby reducing the overall pro-duction yield [5,8]. As a result, understanding the demolding process and its associated stress–strain distribution is critical for ensuring production yield and manufacturing reliability [9,10].

To overcome these problems, additional surface treatment steps, including plasma treatment of molds [11], hydrophilic coating deposition [12], and improvements to punch-type demolding technology [13], are often implemented to enhance the consistency and stability of the demolding process. Plasma treatment enables rapid and large-area processing suitable for mass production; however, it involves high equipment costs. Hydrophilic coatings can improve demolding success rates and surface integrity but may cause lens contamination and surface morphology alterations; thus, precise control of coating materials and deposition parameters is essential. Punch-type demolding involves using a punch pin to apply controlled pressure from beneath or along the side of the mold, inducing slight deformation of the mold to separate the polymerized lens without damaging it. This method requires no additional equipment and can be seamlessly integrated into automated production lines. Therefore, optimizing the punch design has become a key approach for improving demolding efficiency. Although previous studies have proposed various punch-type demolding approaches, most designs focus on modifying the punch tip shape—such as flat-tip, cone-shaped, and protruded punches—to increase localized deformation or reduce stress concentration near the lens edge [14,15,16]. These studies primarily aim to impose direct displacement of the mold bottom along the demolding direction, but they do not introduce structural features that regulate how deformation propagates within the mold. In contrast, the arc-shaped groove design presented in this study introduces a circumferential deformation-guiding structure that redistributes strain more uniformly along the mold sidewall. This feature differs from conventional point-loading punch designs and provides a more controlled mold expansion path, thereby reducing excessive localized deformation that may lead to edge defects. Finite-element analysis (FEA) is a numerical approach used to simulate and predict the behavior of structures, materials, or systems under various loading conditions. This approach involves discretizing a continuous body into multiple smaller elements with defined geometric and material properties; solving the governing equations for displacement, stress, strain, temperature, or other variables; and reconstructing the global response of the system. FEA enables premanufacturing deformation compensation and design optimization for molds and fixtures, improving equipment performance and production yield while substantially reducing development costs [17,18]. However, few studies have used FEA to analyze and optimize punch geometry for improving demolding performance in contact lens manufacturing. Therefore, the present study employed FEA to simulate and evaluate the stress and strain distributions and mold deformation caused by punch designs with different shapes and dimensions. The simulation results were then compared with the results of experimental demolding tests to assess how punch geometry affected process performance. The findings of this research can contribute to enhancing the efficiency and quality of contact lens production and serve as a scientific foundation for the design and optimization of punch-type demolding systems.

## 2. Materials and Methods

### 2.1. Punch-Assisted Demolding Test for Contact Lenses

**Figure 1:**
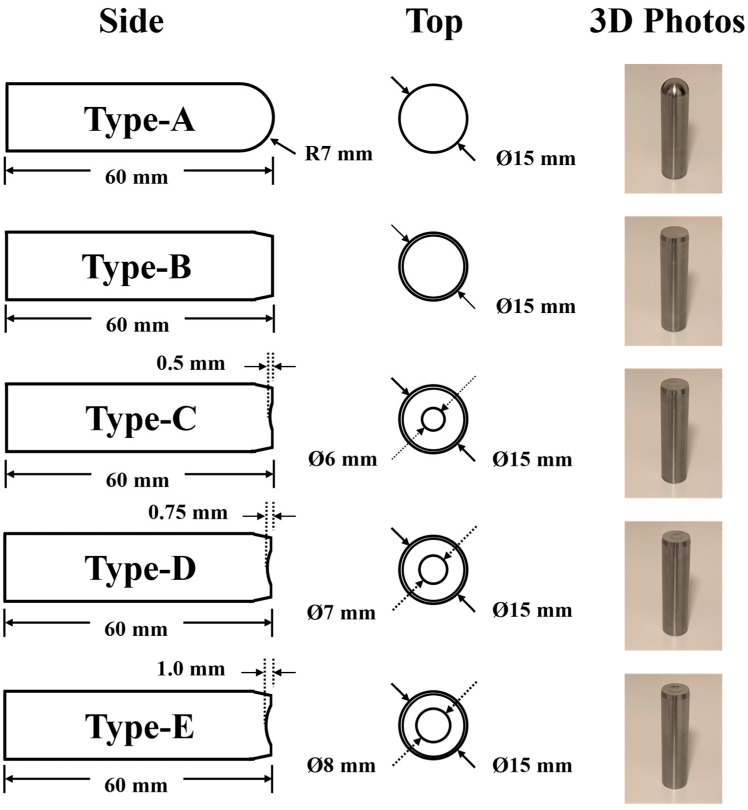
The schematic diagram of five distinct punch pin geometries prepared in this study.

In this study, the upper and lower molds required for fabricating hydrogel contact lenses were produced through the injection molding of polypropylene. The punch used in the fabrication process was made of 304L stainless steel and had a cylindrical shape with a diameter of 15 mm and a length of 60 mm. Five distinct punch head geometries were designed to evaluate the effect of surface morphology on demolding performance. The Type A punch featured a convex surface with a fillet radius of 7 mm, whereas the Type B punch had a flat surface. The Type C, Type D, and Type E punches featured flat surfaces but also incorporated a central arc-shaped groove with a width of 6, 7, and 8 mm and a depth of 0.50, 0.75, and 1.00 mm, respectively, as shown in Figure 1.

**Figure 2:**
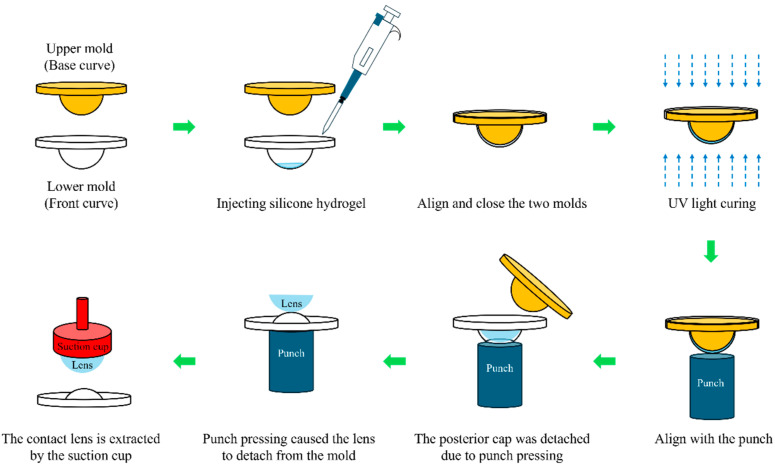
Process flow for the fabrication of contact lenses via the punch-type demolding technique.

All lens fabrication procedures were performed in a Class 100,000 cleanroom under yellow light to prevent premature polymerization, with the environment maintained at 24 ± 0.5 °C and 60 ± 5% relative humidity. Prior to casting, the mold surfaces were cleaned with nitrogen gas to ensure surface cleanliness. The hydrogel precursor solution was then prepared in a mixing vessel by using 2-hydroxyethyl methacrylate (86 wt%; Sigma-Aldrich, Sydney, Australia), ethylene glycol dimethacrylate (0.25 wt%; Aldrich, Sydney, Australia), methyl methacrylate (5.2 wt%; Sigma-Aldrich, Sydney, Australia), methacrylic acid (1.5 wt%; Sigma-Aldrich, Sydney, Australia), N-vinylpyrrolidone (6.05 wt%; Sigma-Aldrich, Sydney, Australia) and Darocur 1173 (1.0 wt%; Ciba-Geigy, Basel, Switzerland). After thorough mixing and continuous stirring for 2 h, the prepared solution was injected into the lower mold cavity. The upper and lower molds were then aligned and assembled. Subsequently, the assembled mold set was exposed to 365 nm ultraviolet light for 10 min at a distance of 10 cm, with an intensity of 15 mW/cm^2^, to initiate photopolymerization, which ultimately resulted in the formation of hydrogel contact lenses. Following polymerization, the lens molds were subjected to punch-assisted demolding tests. During each test, the punch was precisely aligned with the mold center and driven upward by 10 mm at a speed of 1 mm/s, as shown in Figure 2. The demolding test was repeated 100 times to evaluate performance consistency. Throughout the tests, the separation behavior of residual polymer and detachment location were carefully examined. Surface defects and form irregularities on the demolded lenses were further examined using an optical metrology instrument (Optimec Chiltern, Optimec Metrology Limited, Malvern, UK). In addition, to quantify the statistical reliability of the occurrence rates for each defect category, the 95% confidence intervals of the observed proportions were calculated using the Wilson score interval, which provides more accurate bounds for binomial data obtained from repeated trials. The Wilson CI is given by:

(1) $$\mathrm{CI}\;=\;\frac{p+\frac{Z^{2}}{2n}\pm z\sqrt{\frac{p(1-p)}{n}+\frac{z^{2}}{{4n}^{2}}}}{1+\frac{z^{2}}{n}}$$

where *p* is the observed proportion, *n* = 100 is the sample size, and *z* = 1.96 corresponds to a 95% confidence level.

### 2.2. Development of the FEA Model and Fabrication of the Punch Pin

The FEA was performed using the SolidWorks Premium 2020 simulation software to simulate the interaction between the punch and the lower mold during the 10-mm upward displacement. The top surface of the mold was fixed to restrict all translational degrees of freedom, allowing stress and strain responses to occur without geometric displacement. The materials of the punch and mold were set as 304L stainless steel and polypropylene, respectively, and both components were assumed to exhibit isotropic linear-elastic behavior. Based on the mechanical parameters of the demolding process and the geometric characteristics of the adopted molds and punch, a model was developed to analyze the stress and strain behaviors at the contact interface between the punch and the lower molds. The material properties and simulation parameters are summarized in Table 1. Because the mold surface exhibited irregular curvatures, a cubic meshing scheme with an element size of 0.1 mm was employed. This meshing strategy allowed for a detailed assessment of the stress and strain distributions at both the center (point C) and the edge (point E) of the lower mold following the polymerization of the contact lens (diameter: 14.2 mm). The mold–punch interface was modeled using a frictional contact condition with a coefficient of friction μ = 0.20, while the lens–mold interface was represented as a sticky contact to simulate the weak adhesion observed during the actual molding process.

## 3. Results and Discussion

### 3.1. Experimental and Simulation Analysis of Type A and Type B Punches

**Figure 3:**
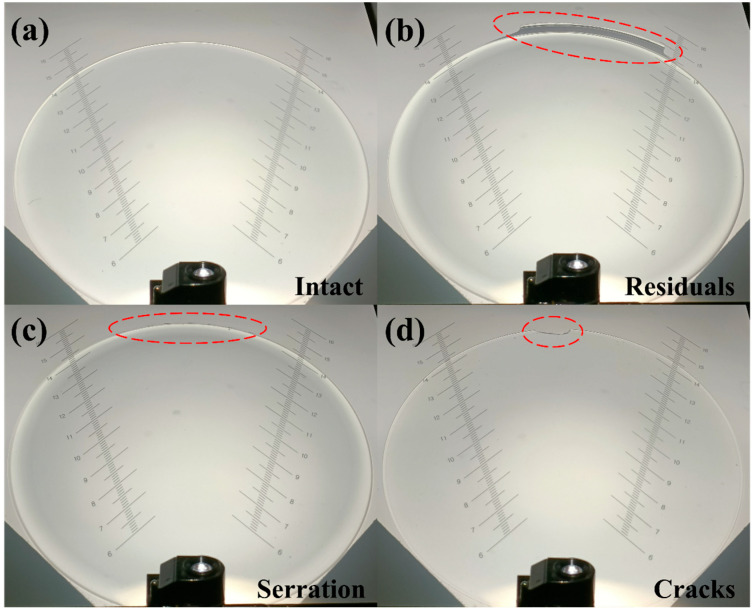
Projection images of different contact lens failures and defects: (**a**) intact, (**b**) residuals, (**c**) serration, and (**d**) cracks.

Table 2 presents the results obtained in the 100 demolding tests conducted with the Type A and Type B punches. The overall process yield was evaluated on the basis of several key indicators, including presence of residual polymer on the lens surface, positional stability, complete detachment, and occurrence of fracturing or deformation, as shown in Figure 3. In addition to the qualitative observations, the analysis using Wilson score confidence intervals further substantiates the distinct demolding behaviors between the two punch designs.

**Figure 4:**
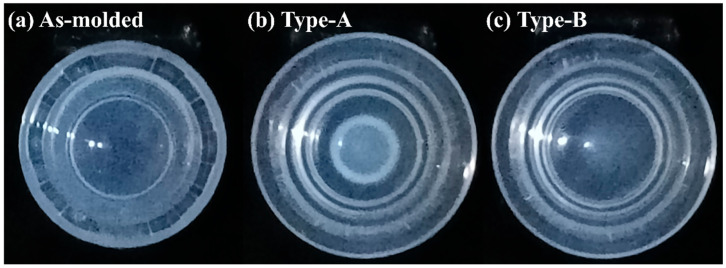
(**a**) As-molded lens; lower molds after demolding using (**b**) the Type-A and (**c**) the Type-B punches.

**Figure 5:**
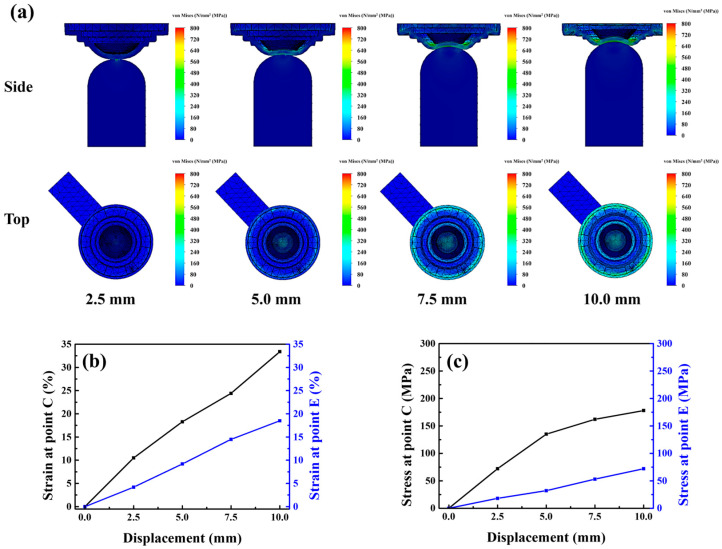
(**a**) Stress model, (**b**) strain curve and (**c**) stress curve of the lower mold simulated under Type-A punch compression.

According to Figure 4b, when demolding was conducted with the Type A punch, the stress-whitening region of the mold was concentrated primarily in its central area (area: 0.43 cm^2^). Numerical simulations revealed that because of the convex geometry of the Type A punch, stress variations primarily occurred at the mold center under different displacement conditions, with the stress at the periphery exhibiting minor changes. As the displacement increased, the central strain increased sharply. When the displacement reached 10 mm, the strain values at points C and E differed substantially, being 33.4% and 18.5%, respectively, as shown in Figure 5a–c.

**Figure 6:**
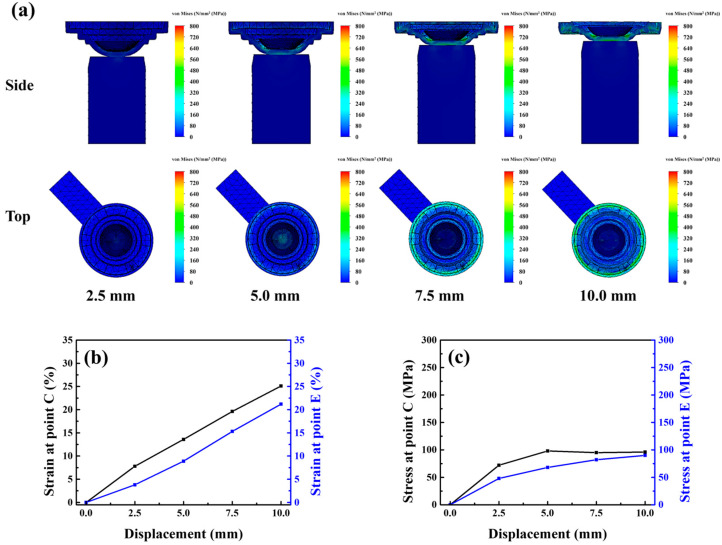
(**a**) Stress model, (**b**) strain curve and (**c**) stress curve of the lower mold simulated under Type-B punch compression.

This concentrated stress field likely promoted fracture initiation in the lens during demolding. Moreover, the rapid disappearance of van der Waals adhesive forces between the lens and the mold caused premature detachment or off-centered positioning, hindering subsequent lens collection. Consequently, the Type A punch had a lens breakage rate of 31% (CI: 22.7–40.6%) and a lens misalignment rate of 28% (CI: 20.1–37.4%), leading to an overall process yield of 62% (CI: 52.2–70.9%). Compared with this punch, The Type B punch, characterized by a flat surface, provided a larger contact area with the mold and generated a broader and lighter stress-whitening region (area: 0.65 cm^2^), as shown in Figure 4c. The Type B punch showed substantially lower lens breakage and misalignment rates, but a higher rate of incomplete lens detachment (25%, CI: 17.5–34.3%), resulting in a moderate yield of 69% (CI: 59.3–77.2%). Simulation analysis Figure 6a–c indicated that the Type B punch produced more uniform stress and strain distributions across the central and peripheral regions than did the Type A punch. When the displacement reached 10 mm, the strains generated by the Type B punch at points C and E were 25.1% and 21.2%, respectively. Thus, although the Type B punch enabled more homogeneous loading than the Type A punch, the uniform strain distribution across the central and peripheral regions prevented the formation of localized peeling initiation points. Consequently, the van der Waals adhesion force remained uniformly distributed, hindering effective lens detachment and subsequent processing. Consistent with this behavior, the confidence intervals indicate that Type B consistently achieves lower structural defects but is more susceptible to adhesion-related detachment failure. These results indicate that the punch must induce an appropriate strain gradient between the central and peripheral regions of the mold to ensure the stable detachment and structural integrity of the lens while maintaining central alignment. This gradient should be gentle to facilitate the gradual dissipation of adhesive force during demolding. To optimize this dissipation behavior, three additional punches were fabricated and analyzed: Type-C, Type-D, and Type-E punches, with groove diameters of 6, 7, and 8 mm, and groove depths of 0.50, 0.75, and 1.00 mm, respectively.

### 3.2. Experimental and Simulation Analysis of Improved Punch Types (C–E)

**Figure 7:**
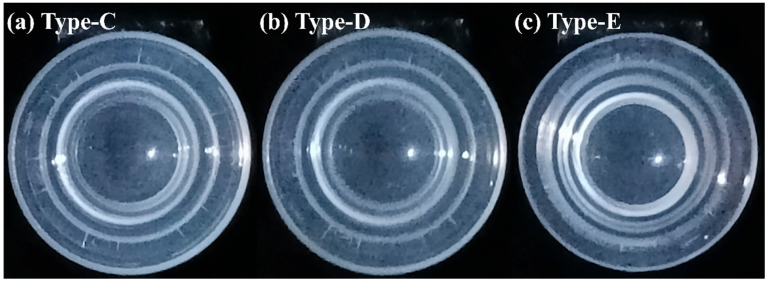
Lower molds after demolding using (**a**) the Type-C, (**b**) the Type-D and (**c**) the Type-E punches.

Table 3 presents the results obtained in the 100 demolding tests conducted and Wilson score confidence intervals with the Type C, Type D and Type E punches. The results indicated that the Type-D punch achieved the highest process yield (82%, CI: 73.3, 88.3) among all fabricated punches. Its stress-whitening region was considerably smaller than those of the Type A and Type B punches and primarily distributed along the outer edge of the mold (area: 0.138), as shown in Figure 7b.

**Figure 8:**
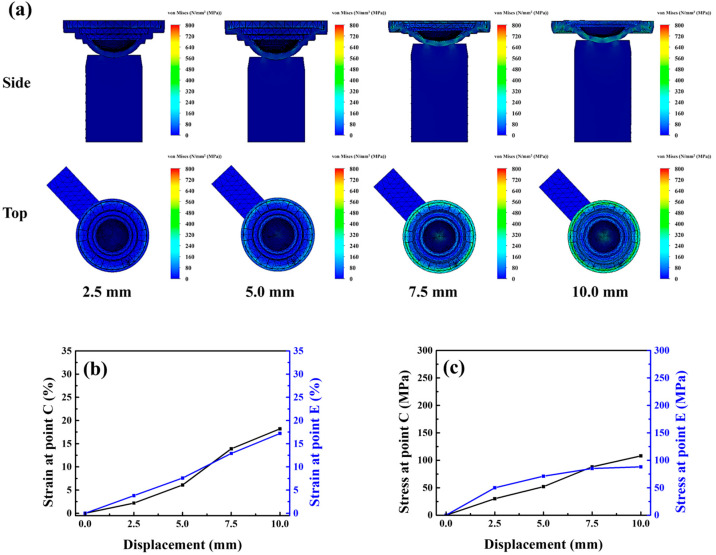
(**a**) Stress model, (**b**) strain curve and (**c**) stress curve of the lower mold simulated under Type-D punch compression.

The simulation results indicated that at the early stage of displacement, stress and strain were concentrated at the periphery of the mold, with limited strain variation occurring at the center. As the displacement increased, a circumferential strain band developed, stabilizing the lens at the mold’s center. When the displacement reached 10 mm, strain gradually transferred inward, causing a two-stage cooperative deformation mechanism between the peripheral and central zones. This mechanism effectively mitigated excessive mold deformation, weakened the adhesive force, and enabled smooth and centered lens detachment. Moreover, the strain at points C and E reached 18.2% and 17.2%, respectively, indicating that the addition of a grooved structure to the flat surface punch further reduced the strain, as shown in Figure 8a–c.

**Figure 9:**
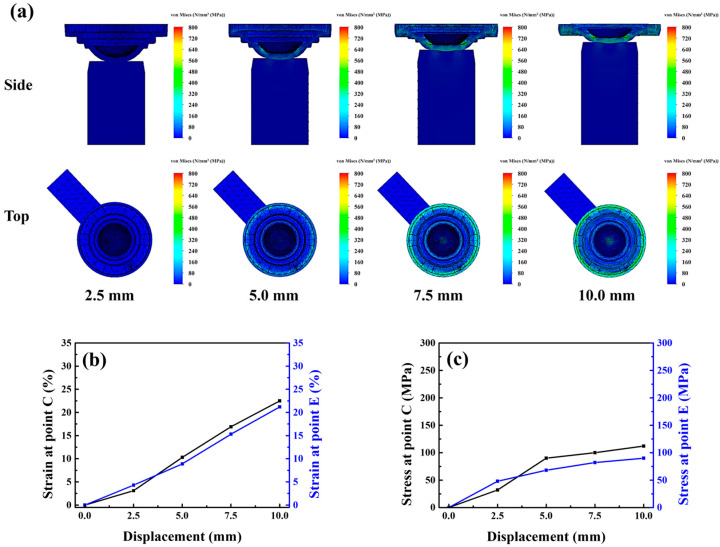
(**a**) Stress model, (**b**) strain curve and (**c**) stress curve of the lower mold simulated under Type-C punch compression.

**Figure 10:**
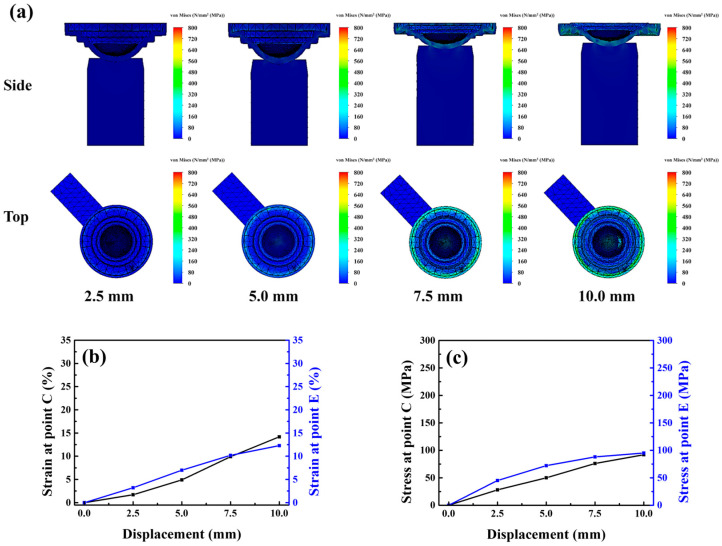
(**a**) Stress model, (**b**) strain curve and (**c**) stress curve of the lower mold simulated under Type-E punch compression.

As shown in Figure 7a, the stress-whitening region of the Type C punch was similar in position and extent to that of the Type D punch (area: 0.148 cm^2^). However, the deviation rate of 13% exhibited a relatively wide confidence interval (CI: 7.7–20.9%), which correspondingly reduced the yield to 78% (CI: 68.9–85.0). The simulation results suggested that a shallower groove caused faster stress transfer from the periphery to the center of the mold. Consequently, when central strain developed, the van der Waals adhesion force began to dissipate earlier, and the insufficient peripheral strain was unable to effectively constrain the lens, leading to greater off-center displacement during demolding. The strain evolution under a displacement of 10 mm is shown in Figure 9a–c. The stress-whitening region of the Type E punch was slightly larger than that of the Type D punch (area: 0.21 cm^2^), as shown in Figure 7c. Although the misalignment rate decreased to 5% (CI: 2.1–11.1%), the relatively broad confidence interval indicates greater variability in alignment outcomes. Moreover, the Type E punch yielded lower demolding efficiency, as residual polymer was observed more frequently. Together, these factors reduced the overall process yield to 73% (CI: 63.5–80.7%). The simulation results revealed that the deeper groove delayed the stress transfer from the periphery to the center of the mold (Figure 10a–c).

Excessive peripheral strain encapsulated the lens, which hindered the dissipation of adhesive force when central strain developed. Furthermore, excessive mold deformation increased edge friction, resulting in the distortion and fracturing of lens edges after demolding. Based on the combined evaluation of experimental results and their correspondence with the simulation data, lens detachment typically occurs when the punch displacement reaches approximately 5–7.5 mm. Correlating this interval with the strain evolution predicted by the FEA model indicates that the strain at points C and E should increase gradually prior to entering this displacement range. Within the detachment interval, the strain at point C must exceed that at point E to promote the progressive reduction of adhesion and establish a stable peeling initiation site. This strain gradient is essential for minimizing damage or edge serration, reducing residual polymer, and preventing misalignment or failure to demold, thereby improving overall demolding performance. In summary, the analytical framework adopted in this study effectively elucidates the correlations between punch geometry, mold deformation behavior, and demolding performance. By integrating finite-element simulation with experimental validation, this framework accurately predicts equivalent stress–strain distributions and quantifies the influences of geometric parameters on process stability and yield. The framework demonstrates strong scalability and can be used for mold design and optimization in other precision molding applications, enhancing product uniformity, process reliability, and manufacturing efficiency.

## 4. Conclusions

The punch-assisted demolding technique is a cost-effective and scalable approach suitable for the large-scale production of contact lenses. In this study, several punch geometries were designed and evaluated through 100 experimental demolding tests each and finite-element simulations to improve punch configuration and enhance overall process yield. The results revealed that the Type D punch (groove diameter: 7 mm, groove depth: 0.75 mm) exhibited the most favorable demolding performance among all fabricated punches. Because of the dispersed initial stress distribution generated by this mold, the lens in it showed gradual strain variations between the central and peripheral regions of the mold. This behavior effectively reduced mold deformation and facilitated the gradual dissipation of van der Waals adhesive forces, thereby allowing the lens to remain centrally positioned after demolding. Comprehensive evaluations of defect occurrence and positional stability indicated that a process yield of 82% was achieved with the Type D punch. Overall, the results of this study indicate that systematic optimization of punch geometry can not only reduce research and development costs but also substantially improve yield and productivity in contact lens manufacturing. The findings of this study highlight the strong potential of using optimized punch-assisted demolding processes for the mass production of contact lenses.

## Figures and Tables

**Table 1:** The material properties and simulation parameters.

Material	Elastic Modulus	Poisson’s Ratio	Density
Punch pin (304L stainless steel)	193 GPa	0.29	8 g/cm^3^
Polypropylene	1.5 GPa	0.42	0.9 g/cm^3^

**Table 2:** Results of lenses obtained from 100 demolding trials conducted with the Type-A and Type-B punches.

Punch Pin	Lens				
	Deviation	Damage or Edge Serration	Residuals	Failure to Demold	Total Yield
Type-A	28%	31%	2%	0%	62%
Type-B	5%	14%	10%	25%	69%

**Table 3:** Results of lenses obtained from 100 demolding trials conducted with the Type-C, Type-D and Type-E punches.

Punch Pin	Lens				
	Deviation	Damage or Edge Serration	Residuals	Failure to Demold	Total Yield
Type-C	13%	15%	2%	0%	78%
Type-D	6%	13%	3%	0%	82%
Type-E	5%	13%	9%	6%	73%

## Data Availability

The original contributions presented in this study are included in the article. Further inquiries can be directed to the corresponding author.
